# Maternal vitamin D insufficiency and risk of adverse pregnancy and birth outcomes: A systematic review and meta-analysis of longitudinal studies

**DOI:** 10.1371/journal.pone.0173605

**Published:** 2017-03-17

**Authors:** A. Kofi Amegah, Moses K. Klevor, Carol L. Wagner

**Affiliations:** 1 Public Health Research Group, Department of Biomedical Sciences, School of Allied Health Sciences, University of Cape Coast, Cape Coast, Ghana; 2 Department of Clinical Nutriton and Dietetics, University of Cape Coast, Cape Coast, Ghana; 3 Medical University of South Carolina Children’s Hospital, Charleston, SC, United States of America; Garvan Institute of Medical Research, AUSTRALIA

## Abstract

**Background:**

Three previous reviews on the association of vitamin D insufficiency in pregnancy with preterm birth (PTB) and stillbirth were limited in scope and deemed inconclusive. With important new evidence accumulating, there is the need to update the previous estimates and assess evidence on other clinically important outcomes such as spontaneous abortion and Apgar score. We conducted a systematic review and meta-analysis to evaluate the quality and strength of the available evidence on the relations between vitamin D nutritional status, and pregnancy and birth outcomes.

**Methods:**

PubMed and Scopus databases were searched from their inception to June, 2015 with no language restrictions imposed. Eighteen longitudinal studies satisfied the inclusion criteria. Random effects model was applied in computing the summary effect estimates and their corresponding 95% confidence intervals.

**Results:**

Serum 25(OH)D levels <75 nmol/l was associated with 83% (95% CI: 1.23, 2.74) and 13% (95% CI: 0.94, 1.36) increased risk of PTB measured at <32–34 weeks and <35–37 weeks, respectively. An inverse dose-response relation was observed for both PTB outcome. Serum 25(OH)D levels <75 nmol/l was also associated with 11% increased risk of spontaneous PTB (<35–37 weeks; RR = 1.11; 95% CI: 0.75, 1.65) with a dose-response relation also noted. Vitamin D insufficiency was not associated with risk of spontaneous abortion and stillbirth (RR of 1.04 [95% CI: 0.95, 1.13] and 1.02 [95% CI: 0.96, 1.09], respectively), as well as short gestational length (ES = -0.24, 95% CI: -0.69, 0.22), and low Apgar score.

**Conclusions:**

We found vitamin D insufficiency to be associated with risk of PTB. Regarding spontaneous abortion and stillbirth, the available evidence suggest no association with low vitamin D levels. The evidence on vitamin D nutrition and Apgar score is conflicting and controversial. Overall, the experimental evidence uncovered was small and weak. Hence, the benefits of vitamin D supplementation during pregnancy should be further evaluated through rigorous intervention studies.

## Introduction

Vitamin D deficiency can result from factors that inhibit its synthesis in the skin, poor dietary consumption and additional factors affecting its absorption or metabolism [1–3]. Vitamin D deficiency is recognized as a public health problem in many parts of the world [3–5], and in some populations, it is common among pregnant women [6,7]. Over the last decade, studies associating vitamin D insufficiency in pregnancy with a wide range of adverse maternal, fetal and neonatal health outcomes have been accumulating in the epidemiological literature, with a number of systematic reviews and meta-analyses [8–14] also attempting to summarize the available evidence. Adverse outcomes linked to vitamin D insufficiency in pregnancy includes pre-eclampsia, gestational diabetes, bacterial vaginosis, small-for-gestational age, low birth weight, impaired fetal skeletal formation and reduced bone mass, and intrauterine growth retardation [10,14,15].

The evidence on preterm birth (PTB) has previously been reviewed by Thorne-Lyman and Fawzi [8] and Harvey et al. [11]. Thorne-Lyman and Fawzi [8] reviewed two trials and found daily supplementation of vitamin D to have no effect on either PTB (<37 weeks; RR = 0.77, 95% CI: 0.35, 1.66) or mean gestational duration (0.17 weeks, 95% CI: -0.16, 0.51). The evidence from observational studies reviewed by the authors was found to be inconsistent. Harvey et al. [11] reviewed seven observational studies and found only one of the studies reporting a significant relationship between maternal vitamin D levels and premature delivery. This review did not report on any intervention studies and concluded that the results of the studies they evaluated were varied but do not support the use of maternal vitamin D supplementation to prevent PTB. De-Regil et al. [9] attempted to review the evidence on vitamin D and stillbirth and uncovered one trial that had investigated the relationship with their results suggesting that vitamin D supplementation is unlikely to prevent stillbirth (RR = 0.17; 95% CI: 0.01, 4.06). The authors concluded that, given the scarcity of data for this outcome, no firm conclusions can be drawn.

A significant number of important new evidence has accumulated since the publication of these reviews. Also, the evidence on other clinically important outcomes such as spontaneous abortion and low Apgar score have not previously been reviewed. These developments call for updating the previous estimates and assessing the state of evidence on these additional outcomes. Furthermore, according to Amegah et al. [16], timely evaluation of methods and results of existing studies should help inform and improve the design of future studies. Our objective is therefore to evaluate the quality and strength of the available evidence on the relations between vitamin D nutrition status, and pregnancy and birth outcomes in order to inform future attempts at revising the WHO guidelines on vitamin D supplementation in pregnancy [17], and propose future research priorities to improve the body of evidence.

## Materials and methods

We conducted and report the study in accordance with the PRISMA (Preferred Reporting Items for Systematic Reviews and Meta- Analyses) guidelines [18].

### Information sources and search strategy

We searched PubMed and Scopus from their inception to the end of June, 2015 with no language restrictions imposed. The search statement applied in the databases was {"Vitamin D" OR "25-Hydroxyvitamin D"} AND {stillbirth OR "fetal death" OR "fetal mortality" OR "perinatal death" OR "perinatal mortality" OR "spontaneous abortion" OR miscarriage OR "preterm birth" OR "preterm delivery" OR "premature birth" OR "apgar score"}. Two independent investigators initially screened the articles for eligibility based on the title and abstract.

### Eligibility criteria and study selection

Articles were considered for inclusion if they satisfied the following: (a) original epidemiologic studies with longitudinal design, (b) conducted in a human population, (c) investigated the relation between vitamin D nutrition status and any of the outcomes listed in the search statement, and (d) assessed vitamin D nutrition status using laboratory methods.

Preterm birth (PTB) was defined as live births before 32 or 37 completed weeks of gestation. Spontaneous abortion was defined as the spontaneous loss of the fetus before 20 weeks of pregnancy whereas stillbirth referred to fetal deaths occuring after 20 week of pregnancy. Vitamin D insufficiency was defined as serum 25(OH)D levels <50 or <75 nmol/l.

Articles were excluded if they were conducted among mothers with non-singleton pregnancies and/or with conditions (including HIV infection, syphillis infection, preeclampsia, gestational diabetes etc.) during pregnancy that places them at high risk for adverse pregnancy outcomes.

Selected articles were retrieved in full and further assessed for eligibility. Studies were included if they either provided effect estimates for the relation between vitamin D nutrition status and the outcomes of interest, or reported proportion of cases of any outcome among vitamin D sufficient and insufficient/deficient mothers.

We also reviewed the reference list of all included studies, and previous related reviews to identify additional eligible studies.

### Data extraction and quality assessment of included studies

Data from eligible studies were extracted independently by the two investigators onto a form. Disagreements during synthesis of the data extracted were resolved through discussion. We contacted authors of included studies for clarifications where needed. Methodological quality of the included studies was assessed by using the original Newcastle-Ottawa Scale (NOS, maximum of 9 stars) for case-control and cohort designs, an adapted NOS (maximum of 6 stars) for cross-sectional *post hoc* analysis, and an adapted Jadad scale that incorporates allocation concealment for randomized trials (maximum of 7 points).

In evaluating the adequacy of confounding control in the included studies, a short list based on *a priori* knowledge of core confounders that needed to be adjusted for in the analysis of such studies was compiled. The short list included maternal age, race/ethnicity and socioeconomic status or their proxies, prepregnancy BMI, parity/gravidity, season and gestational age at blood draw, and smoking status.

### Statistical analysis

We applied the random-effects model, which accounts for both within and between study heterogeneity in computing the summary effect estimates. With regards to studies providing multiple effect estimates, we first combined the effect estimates using fixed-effects model and applied the single effect estimate in the overall meta-analysis. For studies providing estimates for different levels of vitamin D insufficiency, we further conducted a dose response meta-analysis. The effect estimates (risk ratio and odds ratio) reported by the included studies were deemed equivalent owing to the rarity of the outcomes in the study settings and were represented as relative risk (RR). We quantified heterogeneity using the Cochran Q (Χ^2^) test and the *I*^*2*^ statistic with a value > 50% deemed to indicate substantial heterogeneity. Forest plots were also visually assessed. We explored possible sources of heterogeneity by conducting subgroup analysis and meta-regression. We conducted sensitivity analysis by limiting the analysis to very high quality studies; 8 or 9 stars on the original NOS for case-control and cohort studies and 6 or 7 points on the adapted Jadad scale. Publication bias was investigated by visually inspecting funnel plots for asymmetry, and applying the Begg’s and Egger’s tests. We accounted for publication bias using the trim and fill method. Analyses were conducted using Stata version 12.0 (Stata Corporation, College Station, TX, USA).

## Results

**Fig 1:**
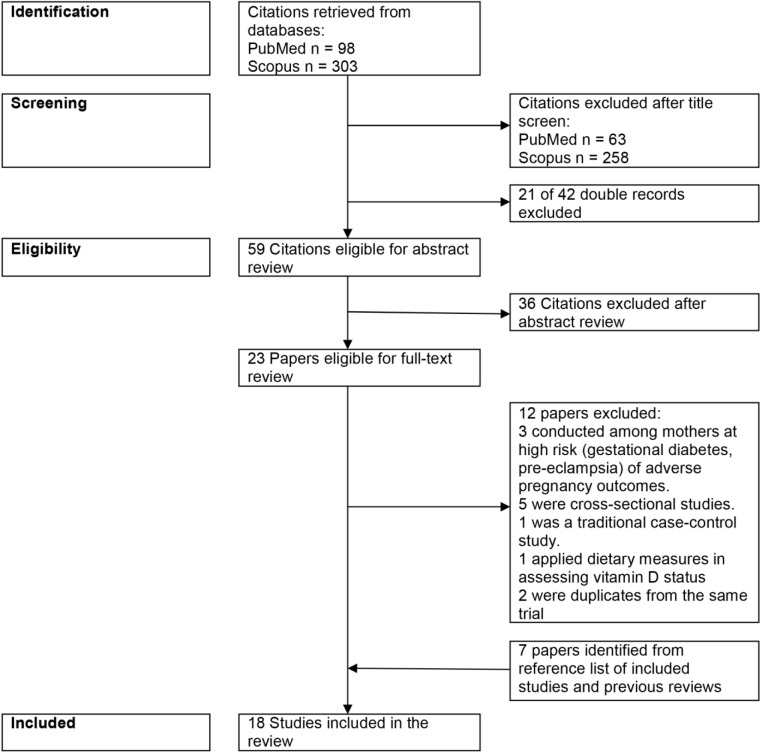
Flow diagram of the study selection process.

A flowchart of the study selection process is depicted in Fig 1. A total of 18 studies were included in the review.

### Characteristics of included studies

The characteristics of included studies are presented in Table 1. Four studies [19, 20, 21, 22] were randomized controlled trials (RCTs).Two studies [23, 24] were *post hoc* analyses of RCT. Prospective cohort design was applied by six studies [25, 26, 27–29, 30]. One study [31] was a restrospective cohort. Four studies [24, 32, 33, 34] adopted a nested case-control design, including one of the RCT *post hoc* analysis [24]. Two studies [35, 36] applied a case-cohort design. Eight studies [21, 24, 23, 31, 32, 34, 35, 36] were conducted in the United States of America. Four studies [20, 25, 27, 30] were conducted in Europe, with two [27, 30] conducted in Spain and the remaining two undertaken in Denmark [25] and United Kingdom [20]. Two studies [28, 33] were conducted in Australia. Four studies were conducted in Asia [19, 22, 26, 29] with one study each undertaken in India [19], Pakistan [22], South Korea [29], and China [26].

**Table 1:** Characteristics of included studies.

First author, Year (Reference No.)	Location and period	Design	Population and sampling	Vitamin D assay method, time and reference level	Outcomes and measurement	Confounding control	Main results	Quality score
Baker, 2011 (34)	• North Carolina, USA • 11/2004–7/2009	Nested case-control	40 cases and 120 race/ ethnicity-matched controls were selected from an eligible cohort of 4225 women delivering at the University of North Carolina	• LC-MS • 11–14 weeks of gestation • ≥75 nmol/L	• sPTB (≥23^0^/_7_ and ≤34^6^/_7_ weeks) • GA determined by LMP method and ultrasound estimates	Maternal age, insurance status, BMI, GA at serum collection, season of blood draw	**Adjusted OR (95% CI)**	8/9
							• 50–74.9 nmol/L: 0.87 (0.34, 2.25) • <50 nmol/L: 0.82 (0.19, 3.57) • Combined (<75 nmol/L): 0.86 (0.39, 1.89)	
Bodnar, 2015 (36)	• Pittsburgh, Pennsylvania • 1999–2001, 2003, 2007–2010	Case–cohort	• 2327 randomly sampled singleton liveborn neonates delivered at the Magee-Womens hospital formed the subcohort of which 204 were cases. • Subcohort was augmented with 922 cases in the eligible cohort giving a total of 1126 cases	• LC-MS • 20 weeks of gestation or earlier • ≥75 nmol/L	• PTB (<37 and <34 weeks) and sPTB (<37 weeks) • GA at delivery determined using best obstetric estimate based on comparison of LMP and ultrasound dating	Maternal race and ethnicity, prepregnancy BMI, parity, maternal education, marital status, smoking status, season and gestational age of blood sampling, assay batch, and year of delivery.	• PTB incidence declined significantly with increasing 25(OH)D levels (p<0.01) • **Adjusted RR (95% CI)** • PTB (<37 weeks): <50 nmol/L, 1.8 (1.3, 2.6); 50–74.9 nmol/L, 1.4 (1.1, 1.8); Combined (<75 nmol/L) 1.51 (1.22, 1.85) • sPTB (<37 weeks): <50 nmol/L, 1.8 (1.2, 2.7); 50–74.9 nmol/L, 1.3 (0.9, 1.8); Combined (<75 nmol/L), 1.49 (1.15, 1.94) • PTB (<34 weeks): <50 nmol/L, 2.1 (1.3, 3.6); 50–74.9 nmol/L, 2.2 (1.4, 3.4); Combined (<75 nmol/L), 2.16 (1.54, 3.01)	9/9
Bodnar, 2014 (35)	• 12 medical centers in USA • 1959–1965	Case-cohort	• 2,629 pregnancies from a cohort of deliveries of singleton liveborn infants at 26–42 weeks gestation formed the subcohort of which 104 were cases. • Subcohort was augmented with 735 cases in the eligible cohort giving a total of 839 cases of which 767 were sPTB	• LC-MS • 26 weeks of gestation or earlier • <30 nmol/L	• sPTB (26 - <35 weeks) • GA determined by LMP method • Strict GA and birth weight limits used to reduce misclassification • Placental pathological examinations conducted	Maternal age, socioeconomic position, parity, marital status, pre-pregnancy BMI, smoking during pregnancy, trimester of entry to prenatal care, GA and season at blood sampling, study site	**Adjusted RR (95% CI)**	9/9
							• Non-white mothers: 30 - <50 nmol/L, 0.78 (0.62, 0.99); 50 - <75 nmol/L, 0.64 (0.48, 0.86); ≥75 nmol/L, 0.66 (0.44, 0.98) • White mothers: 30 - <50 nmol/L, 1.0 (0.64, 1.7); 50 - <75 nmol/L, 1.1 (0.66, 1.7); ≥75 nmol/L, 1.0 (0.61, 1.7)	
Flood-Nichols, 2015 (31)	Madigan, USA	Retrospective cohort	235 healthy, nulliparous women who received prenatal care and delivered at Madigan Army Medical Center were recruited	• ELISA and confirmed by LC-MS • 5–12 weeks of gestation • ≥30 ng/ml	• sPTB (<37 weeks) and SaB • GAs were confirmed with first or second trimester ultrasound, and verified by chart review at study completion	BMI, season, ethnicity, tobacco use	**Adjusted OR (95% CI)**	7/9
							• sPTB: 0.78 (0.17, 3.55) • SaB: 0.65 (0.18, 2.28)	
Rodriguez, 2014 (30)	• Valencia, Sabadell, Asturias and Gipuzkoa, Spain • 2003–2008	Prospective cohort	• 2358 women recruited during the first prenatal visit (10–13 weeks of gestation) in the main public hospital or health centre in the four study areas. • Response rate was 89%	• HPLC and validated • Mean time of 13.5 weeks of gestation • <20 ng/ml	• PTB (<37 weeks) • GA determined by LMP with early ultrasound of the crown-rump length used when the difference with the LMP was ≥7 days (12% of newborns)	Child’s sex, parity, maternal social class, education, age at delivery, smoking during pregnancy, overweight, alcohol consumption during pregnancy, area of study	• No significant difference in mean 25(OH)D3 levels among mothers delivering preterm and term births (GM: 28.79 ng/ml [95% CI: 26.78, 30.96] vs. 28.22 ng/ml [95% CI: 27.77, 28.67], p value = 0.592) • **Adjusted RR (95% CI)** • 20–29 ng/ml: 0.98 (0.52, 1.85) • ≥30 ng/ml: 1.08 (0.75, 1.67) • Combined (<50nmol/L): 0.95 (0.68, 1.33) • Quartiles analysis showed decreased risk at higher quartiles	9/9
Sablok, 2015 (19)	New Delhi, India	Randomized controlled trial	• 180 primigravidae with singleton pregnancy were recruited at the Safdarjung Hospital and assigned to control group (n = 60) and intervention group (n = 120) who received vitamin D in doses depending on the levels estimated at enrollment • Losses to follow up were 3 and 12, respectively	• ELISA • 14–20 weeks of gestation (Intervention group) and delivery (both groups)	• PTL and Apgar score (5 minutes) • Method for determining GA not reported		• Median 25(OH)D levels among intervention mothers was 65 nmol/l post-supplementation compared to 38 nmol/l pre-supplementation and 24 nmol/l in control mothers • Positive correlation between maternal vitamin D status and apgar score (r = 0.325, p = 0.000). • A significant decrease in PTL/PTB incidence was noted in the intervention group (p = 0.02)	2/7
Schneuer, 2014 (33)	• New South Wales, Australia • 10/2006–9/2007	Nested case-control	Cases (PTB = 388, SaB = 39, Stillbirth = 33) and 3714 controls were selected from an eligible cohort of 5109 women who were attending first-trimester Down syndrome screening	• Automated immunoassay (CLIA) • 10–14 weeks of gestation • >75 nmol/L	• PTB (<37 weeks), Early PTB (<34 weeks), sPTB, SaB (10–20 weeks) and Stillbirth (>20 weeks) • Method for determining GA not reported	Maternal age, parity, smoking during pregnancy, maternal weight, previously diagnosed hypertension, previously diagnosed diabetes, season at sampling, country of birth, socioeconomic disadvantage	Unadjusted OR (95% CI)*	9/9
							• All PTB: 1.14 (0.88, 1.49) • All early PTB: 1.42 (0.86, 2.36) • SaB: 0.89 (0.42, 1.86) • Stillbirth: 1.25 (0.52, 3.00)	
Thorp, 2012 (24)	• 13 centers in USA • 1/2005–10/2006	Post Hoc analysis (RCT of daily EPA and DHA supplement) using a nested case-control design strategy	Controls (n = 134) and cases (n = 131) matched on race/ethnicity and study site in 1:1 ratio were respectively selected from intervention (n = 434) and placebos (n = 418) patients that delivered at ≥37 weeks of gestation	• LC-MS • 16–22 weeks of gestation and during follow-up at 25–28 weeks of gestation in a subset of 80 cases and 88 controls • ≥50 nmol/l	• Recurrent PTB (<35 weeks) and Very early PTB (<32 weeks) • Method for determining GA not reported	Race/ethnicity, study center, maternal age, number of prior preterm deliveries, smoking status, BMI, season when blood was drawn, treatment group and fish intake	• No significant difference in baseline mean 25(OH)D concentration among cases and controls (70.7 nmol/l [SD: 30.7] vs. 72.7 [SD: 32.6], p = 0.61) • **Adjusted OR at baseline (95% CI)** • All women (<50 nmol/l): 0.80 (0.38, 1.69) • African American women (<50 nmol/l): 1.09 (0.45, 2.67) • Follow-up visit analysis produced similar null findings • No association was observed between low vitamin D status and very early PTB • Quartile analysis revealed slight decreased odds at higher quartiles	9/9
Wagner, 2015 (23)	• South Carolina, USA • 1/2004–8/2010	Post Hoc analysis of two combined RCTs of vitamin D supplementation using a cross-sectional design strategy	A total of 487 women were enrolled into the two trials	• RIA • <16 weeks gestation, second trimester (16–26 weeks gestation) and third trimester (≥27 weeks to delivery) • <100 nmol/L	• PTB (<37 weeks) • GA determined by LMP or obstetrical estimate at the time of first visit if mother was unsure and confirmed at 20-week fetal ultrasound	Study and race/ethnicity	**Third trimester adjusted OR** • ≥100 nmol/L: 0.53 (p = 0.08) • Quartile analysis revealed much greater decreased odds at higher quartiles in each of the trimesters	4/6
Hollis, 2011 (21)	• Charleston, South Carolina, USA • 1/2004–7/2009	Randomized double-blinded controlled trial	• 502 women with confirmed singleton pregnancy were enrolled and randomized to receive one of three vitamin D_3_ treatment regimens (400, 2000 or 4000 IU/day) based on their baseline levels. • Women exiting the study before receiving the intervention and those lost to follow up was 5 and 147, respectively	• RIA • ≤16 weeks of gestation, 1 month before delivery and at delivery	• GL and SaB • GA determined by LMP or obstetrical estimate at the time of first visit if mother was unsure and confirmed at 20-week fetal ultrasound		• Mean (SD) GL was 38.6 (2.2), 38.8 (1.8) and 39.1 (1.8) weeks for treatments groups 400 IU, 2000 IU and 4000 IU daily, respectively (Mean differences was not significant, p = 0.17) • Mean baseline 25(OH)D levels of women who experienced SaB (50.5±23.3 nmol/L, n = 23) was lower than those who delivered a live-birth (57.8±24.4 nmol/L, n = 350); the difference was not statistically significant	7/7
Morley, 2006 (28)	• Geelong, Australia • 4/2002–9/2003	Prospective cohort	374 women were recruited at the Geelong Hospital antenatal clinic	• RIA • < 16 weeks of gestation and follow up at 28–32 weeks gestation	• GL • Method for determining GA not reported	Infant sex, maternal height, birth order (first child), smoking during pregnancy, and season when blood sample was taken	• Mean (SD) GL was 38.7 (1.5) and 39.5 (1.9) weeks among women with low (<28 nmol/l) and normal (≥28 nmol/l) 25(OH)D levels, respectively (Adjusted mean difference = -0.8, 95% CI: -1.4, 0.2) • A unit increase in log_2_ 25(OH)D was associated with a 0.3 week (95% CI: 0.07, 0.6) increase in GL	6/9
Hossain, 2014 (22)	• Karachi, Pakista • 9/2010–5/2012	Randomized controlled trial	193 women with singleton pregnancy attending the outpatient obstetric clinic at the Civil Hospital were assigned to a control group which received routine care regimen, and an intervention group which received vitamin D in addition to the routine care regimen	• CLIA • ≤20 weeks of gestation and delivery	• GL, PTB (<37 weeks), Intrauterine fetal death and Apgar score • Method for determining GA not reported		• No significant difference in mean GL between control and intervention group (37.66 [SD: 2.0] vs. 37.56 [SD: 1.9], p = 0.29) • Vitamin D supplementation had no effect on risk of PTB (p = 0.67) • Babies of intervention mothers recorded significantly higher 1-minute and 5-minute Apgar scores compared to babies of control mothers (7.10 [SD: 0.66] vs. 6.90 [SD: 0.55], p = 0.03 and 8.53 [SD: 0.68] vs. 8.33 [SD: 0.81], p = 0.05 respectively)	2/7
Moller, 2012 (25)	• Aarhus, Denmark • 10/2006–1/2008	Population-based prospective cohort	153 healthy Caucasian women aged 25–35 years with immediate pregnancy plans were recruited of which 92 women conceived and were followed	• LC-MS • Before pregnancy and three follow-up visits (weeks 11±2, 22±1 and 35±2)	• GL, SaB and Apgar score • GA determined by LMP or ultrasound	Age of woman, height, weight changes during pregnancy, smoking status, parity, sex of infant, season of birth, time of year of gestation, time of year of giving birth (summer vs winter time), daily calcium intake	• No significant difference in baseline 25(OH)D levels among women who had SaB and those who did not (n = 8, median: 54 nmol/l [IQR: 38, 62] vs. 62 nmol/l [IQR: 49, 72], p = 0.14). • Women who had SaB after 10th week of pregnancy had lower 25(OH)D levels at the 2nd visit compared with those who did not (36 nmol/l [Range: 35, 54] vs. 65 nmol/l [Range: 24, 111], p = 0.03). • 25(OH)D levels did not predict Apgar score (94% and all babies respectively had 1- and 5-minute Apgar score of >8). • 25(OH)D levels did not affect GL (p > 0.09)	7/9
Fernandez-Alonso, 2012 (27)	• Almería, Spain • 5/2009–4/2010	Prospective cohort	466 pregnant women attending their first prenatal visit at the outpatient clinic of the Torrecárdenas Hospital were recruited	• Electro-CLIA • 11–14 weeks of gestation, and third trimester (36–39 weeks) in a subset of participants. • ≥75 nmol/l	• Apgar score and PTB (21−36^6^/_7_ weeks) • Method for determining GA not reported		Unadjusted OR (95% CI)*	6/9
							• 1-minute Apgar score of <7: 1.40 (0.43, 4.54) • PTB: <75 nmol/l, 1.12 (0.53, 2.37); 50–74.9 nmol/l, 1.2 (0.54, 2.69); <50 nmol/l, 0.97 (0.36, 2.58)	
Wetta, 2014 (32)	• Birmingham, Alabama, USA • 2007–2008	Nested case-control	90 cases and 177 controls were selected from an eligible cohort of women with singleton pregnancies at the University of Alabama at Birmingham Department of Obstetrics and Gynecology	• LC-MS • 15–21 weeks of gestation • ≥30 ng/mL	• GL and sPTB (<35^0^/_7_ weeks) • GA determined by ultrasound	Age, race, parity, weight, prior preterm birth and season of specimen collection	• Mean serum 25(OH)D levels was not significantly different between controls and cases (28.6 [SD: 12.6] vs. 28.8 [SD: 13.2], p = 0.92). • Mean GA was significantly different between controls and cases (39.4 ± 0.5 vs. 30.1 ± 3.2, p < 0.001) • **Adjusted OR (95% CI)** • Vitamin D insufficiency (<30 ng/mL): 0.8 (0.4, 1.4) • Vitamin D deficiency (<15 ng/mL): 1.3 (0.6, 3.0)	8/9
Yu, 2009 (20)	• London, UK • 4/2007–11/2007	Randomized Controlled Trial	180 pregnant women from four ethnic populations (Indian Asians, Middle Eastern, Black and Caucasian; n = 45 each) visiting St Mary’s Hospital were recruited and randomized within each ethnic group to two treatment groups and a control group	• Assay method not reported • 27 weeks of gestation and at delivery	• GL and Stillbirth • Method for determining GA not reported		• No significant difference in GA at delivery between intervention and control groups • Control group recorded the only one unexplained stillbirth at 41 weeks	3/7
Choi, 2015 (29)	• An Urban area of South Korea • 4/2012–9/2013	Prospective cohort	220 pregnant women were recruited	• LC-MS/MS • First prenatal visit in any trimester of pregnancy • ≥ 20 ng/mL	• PTB (<37 weeks) • GA determined by LMP and ultrasound	Age, trimester and seasons of blood draw and 25(OH)D measurements, education level, job, type of current pregnancy, concurrent pregnancy status, gravity, parity, previous or concurrent medical history, and gynecological disease history	Adjusted OR (95% CI): 0.699 (0.144–3.402)	6/9
Zhou, 2014 (26)	• Guangzhou city, China • 9/2010–8/2011	Prospective Cohort	• All pregnant women visiting a teaching Hospital during the period were invited to participate. • Response rate was 96.1% (n = 2960). • 1953 women included in pregnancy outcome analysis	• Electro-CLIA • 16–20 weeks of gestation • ≥ 30 ng/mL	• PTB (<37 weeks), SaB (<20 weeks), Intrauterine fetal death (>20 weeks), and 1 and 5- minute Apgar score • Method for determining GA not reported	Maternal age, systolic/ diastolic pressure, prepregnancy BMI and serum calcium	**Adjusted OR (95% CI)**	7/9
							• PTB: 1.04 (1.02, 1.06) • SaB: 1.04 (0.95, 1.13) • Fetal death: 1.02 (0.96, 1.09) • No significant difference in 1 and 5- minute Apgar score between the three serum 25(OH)D levels (≤20 ng/ml, 21–29 ng/ml and ≥30 ng/ml, p = 0.673 and 0.497, respectively)	

Abbreviations: 25(OH)D, 25-hydroxyvitamin D; BMI, Body mass index; CLIA, Chemiluminescence Immunoassay; DHA, Docosahexaenoic acid; ELISA, Enzyme-linked immunosorbent assay; EPA, Eicosapentaenoic acid; GA, Gestational age; GL, Gestational length; HPLC, High-performance liquid chromatography; IQR, Interquartile range; LC-MS, Liquid chromatography-tandem mass spectrometry; LMP, Last menstrual period; OR, Odds ratio; PTL, Preterm labour; RD, Risk difference; RIA, Radioimmunoassay; RR, Risk ratio/Relative risk; SaB, Spontaneous abortion; SD, Standard deviation; SE, Standard error; sPTB, Spontaneous preterm birth

*Computed from data presented in manuscript.

All the included studies, except one [20] used one of five different techniques—enzyme-linked immunosorbent assay (ELISA), chemiluminescence immunoassay (CLIA), radioimmunoassay (RIA), liquid chromatography-tandem mass spectrometry (LC-MS) and high-performance liquid chromatography (HPLC) to measure vitamin D metabolite in serum or plasma. Yu et al. [20] did not indicate the method used for the vitamin D assay. The studies also used varying cut-offs for vitamin D insufficiency/deficiency ranging from serum 25(OH)D levels of <50 to <75 nmol/l.

Gestational length was ascertained by all of the included studies with eleven of the studies using varying gestational age cut-offs ranging from <32 to <37 completed weeks to investigate PTB occurence. Sablok et al. [19] investigated preterm labor (PTL). Seven [21, 23, 25, 29, 30, 34, 36] of the studies that investigated gestational length complemented the last menstrual period (LMP) method with fetal ultrasound in estimating gestational age. One study [35] complemented the LMP method with birth weight limits, and also conducted placental pathological examinations. Two studies [31, 32] relied on ultrasound method solely. In the remaining eight studies, there was no mention of the method used in estimating gestational age. Spontaneous abortion and stillbirth was investigated by five [21, 25, 26, 31, 33] and four [20, 22, 26, 33] studies, respectively. Five studies [19, 22, 25, 26, 27] investigated Apgar score.

### Methodological quality of included studies

There was the potential for selection bias in the retrospective cohort study due to the convenience sampling approach adopted for recruiting participants. Selection of participants into the prospective cohort studies was unrelated to the exposure and hence selection bias is unlikely in these studies. However, three of the studies [27–29] were relatively small, and this raises questions as to whether the cohort is a representative sample of the reference population. Selection bias was minimized in the nested case-control studies as the cases and controls originated from the same reference population and should be more representative. All four RCTs and the other two that underwent *post hoc* analysis also applied convenience sampling techniques in recruiting participants from health facilities. The extent to which these samples were representative of their reference populations were again not clear and hence, the potential for selection bias in these studies.

In all the trials, the randomization procedures were indicated in the report and were appropriate. One trial [21] provided a description in their report that suggested allocation concealment. There was also no mention of blinding in two of the trials [19, 22]. Yu et al. [20] indicated not blinding study personnel and participants to the treatment assignment. Two of the trials [20, 21] provided information on withdrawals and losses to follow-up in each treatment group.

The potential for information bias was unlikely in all the studies due to the use of laboratory methods in assessing vitamin D nutrition status. For all the included studies, the outcomes were objectively ascertained at health facilities. Of the studies that ascertained gestational length, the potential for outcome measurement bias was likely to be minimised in studies that estimated gestational age by using ultrasound method, or complemented the LMP method with other method(s). In studies that did not mention the method used for estimating gestational age in their report, assessing validity of the outcome measures was impossible. Of the studies that ascertained stillbirth and spontaneous abortion, only two [20, 33] provided a case definition in their report.

All except three of the RCTs [19, 21, 22] and one prospective cohort study [27] adjusted for a range of potential confounders in the analysis, including demographic and socioeconomic characteristics, maternal nutritional and healthcare characteristics, smoking and alcohol consumption during pregnancy, obstetric history, season of specimen collection and birth, and newborn characteristics. One study [35] further conducted a probabilistic bias analysis to assess unmeasured confounding by physical activity, fish intake and skin color/melanin content. Based on our *a priori* criteria, of the included studies that adjusted for potential confounders, confounding control was considered adequate in five studies [24, 33, 30, 35, 36].

Overall, applying the respective study quality assessment scales, eight studies [21, 24, 30, 32–36] were classified as very high quality (RCTs—6 or 7 points; case-control/cohort—8 or 9 stars), three studies [25, 26, 31] as high quality (RCTs—5 points; case-control/cohort—7 stars), four studies [20, 27–29] as satisfactory quality (RCTs—3 or 4 points; case-control/cohort—5 or 6 stars) and three studies [19, 22, 23] as low quality (RCTs—<3 points; <5 stars for both case-control/cohort and post hoc cross-sectional analysis).

### Summary-effect estimates and evidence of statistical heterogeneity

#### Preterm labour and preterm birth

One intervention study [19] provided evidence on the relation of vitamin D status with PTL. This study used a *per* protocol analysis and found mothers in the control group to have 193% increased risk of PTL.

On the relation of vitamin D status with PTB, the only intervention study identified [22], which also conducted a *per* protocol analysis found control mothers to have 22% decreased risk of PTB. The association was, however, not statistically significant. Of the evidence from observational studies, Wagner et al. [23] reported a 47% decreased odds of PTB among mothers with third trimester serum 25(OH)D concentration of ≥100 nmol/L (Adjusted OR = 0.53, p = 0.08) compared to those with concentrations < 100 nmol/L. Quartile analysis also revealed much greater decreased odds of PTB at higher quartiles in each of the trimesters of pregnancy.

**Fig 2:**
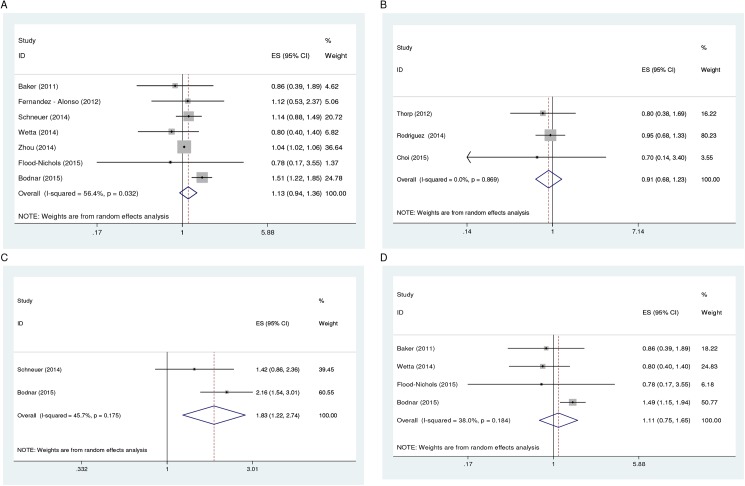
**Forest plot showing the association of PTB (<35–37 weeks) with vitamin D insufficiency at serum levels of <75 nmol/l (A) and <50 nmol/l (B), and PTB (<32–34 weeks) and spontaneous PTB (<35–37 weeks) at serum levels of <75 nmol/l (C and D, respectively).** Abbreviations: ES, Effect Size; CI, Confidence Interval.

Ten studies, all observational design, provided estimates for the quantitative review of the relation of vitamin D insufficiency with PTB. Of these studies, seven [26, 27, 31–34, 36] applied a serum 25(OH)D cut-off level of 75 nmol/l, with the meta-analysis revealing maternal levels below this cut-off to be associated with 13% increased risk of PTB measured at <35–37 weeks (Summary RR = 1.13; 95% CI: 0.94, 1.36). Even though the confidence interval includes the null value, it is narrow and suggests that, the effect estimate is statistically stable and less influenced by random error. Moderate evidence of statistical heterogeneity (*I*^*2*^ = 56.4%) was observed among the seven studies meta-analyzed (Table 2, Fig 2A). The results from the sensitivity analyses was attenuated with substantial evidence of heterogeneity observed (RR = 1.18; 95% CI: 0.90, 1.55; *I*^*2*^ = 52.3%; n = 4). Four of these seven studies [27, 33, 34, 36] provided estimates for dose-response meta-analysis. An inverse relationship was noted, with decreasing serum 25(OH)D levels found to be associated with a statistically significant increased risk of PTB (Summary RR of 1.24 [95% CI: 1.04, 1.49] and 1.36 [95% CI: 1.04, 1.78] for serum 25(OH)D levels of 50–75 nmol/l and <50 nmol/l, respectively). Two of the studies [33, 36] further provided estimates for PTB measured at <32–34 weeks with serum 25(OH)D levels <75 nmol/l found to be associated with a much higher statistically significant increased risk (Summary RR = 1.83; 95% CI: 1.23, 2.74). Moderate evidence of statistical heterogeneity was observed in the analysis (Table 2, Fig 2C). An inverse dose-response relationship was also observed for PTB measured at <32–34 weeks (Summary RR of 1.70 [95% CI: 0.98, 2.95] and 1.86 [95% CI: 1.28, 2.68] for serum 25(OH)D levels of 50–75 nmol/l and <50 nmol/l, respectively).

**Table 2:** Summary relative risk (RR) and effect size (ES) for the relation of vitamin D insufficiency with pregnancy and birth outcomes.

		Random-effects model		Heterogeneity		
Outcome	No. of studies	RR/ES	95% CI	Cochran Χ^2^	p value	*I*^*2*^ (%)
Gestational length	3	-0.24	-0.69, 0.22	8.25	0.016	75.8
PTB (<35–37 weeks, <75 nmol/l)^a^	7	1.13	0.94, 1.36	13.76	0.032	56.4
PTB (<35–37 weeks, 50–75 nmol/l)^a^	4	1.24	1.04, 1.49	2.23	0.525	0.0
PTB (<35–37 weeks, <50 nmol/l)^a^	4	1.36	1.04, 1.78	3.55	0.315	15.5
PTB (<32–34 weeks, <75 nmol/l)^a^	2	1.83	1.23, 2.74	1.84	0.175	45.7
PTB (<32–34 weeks, 50–75 nmol/l)^a^	2	1.70	0.98, 2.95	2.48	0.116	59.6
PTB (<32–34 weeks, <50 nmol/l)^a^	2	1.86	1.28, 2.68	0.47	0.491	0.0
sPTB (<35–37 weeks, <75 nmol/l)^a^	4	1.11	0.75, 1.65	4.84	0.184	38.0
sPTB (<35–37 weeks, 50–75 nmol/l)^a^	2	1.24	0.90, 1.72	0.61	0.434	0.0
sPTB (<35–37 weeks, <50 nmol/l)^a^	2	1.69	1.11, 2.58	1.03	0.311	2.5
PTB (<35–37 weeks, <50 nmol/l)^b^	3	0.91	0.68, 1.23	0.28	0.869	0.0
Spontaneous abortion (<75 nmol/l)^a^	3	1.04	0.95, 1.13	0.69	0.710	0.0
Stillbirth (<75 nmol/l)^a^	2	1.02	0.96, 1.09	0.21	0.650	0.0

Abbreviations: CI, confidence interval; RR, summary relative risk; ES, effect size; PTB, preterm birth; sPTB, spontaneous preterm birth.

^a^25(OH)D levels ≥75 nmol/l served as reference.

^b^25(OH)D levels ≥50 nmol/l served as reference.

The remaining three studies [24, 29, 30] applied a serum 25(OH)D cut-off level of 50 nmol/l, with the meta-analysis revealing no association between maternal levels below this cut-off and risk of PTB measured at <35–37 weeks (Summary RR = 0.91; 95% CI: 0.68, 1.23). No evidence of statistical heterogeneity was observed among the three studies meta-analyzed (Table 2, Fig 2B). The sensitivity analysis produced very similar results (RR = 0.92; 95% CI: 0.68, 1.25; *I*^*2*^ = 0.0%; n = 2). Four studies [31, 32, 34, 36] provided estimates for spontaneous PTB measured at <35–37 weeks, with the meta-analysis showing serum 25(OH)D levels <75 nmol/l to be associated with 11% (Summary RR = 1.11, 95% CI: 0.75, 1.65) increased risk of spontaneous PTB (Table 2, Fig 2D). Again, even though the confidence interval includes the null value, the effect estimate is statistically stable and less influenced by random error owing to the narrow confidence interval. Moderate evidence of statistical heterogeneity was noted in the meta-analysis (*I*^*2*^ = 38%). Two [31, 36] of these studies provided estimates for a dose-response meta-analysis with an inverse relationship also noted (Summary RR of 1.24 [95% CI: 0.90, 1.72] and 1.69 [95% CI: 1.11, 2.58] for serum 25(OH)D levels of 50–75 nmol/l and <50 nmol/l, respectively). The sensitivity analysis produced very similar results (RR = 1.11, 95% CI: 0.70, 1.75; *I*^*2*^ = 54.6%; n = 3).

#### Spontaneous abortion

**Fig 3:**
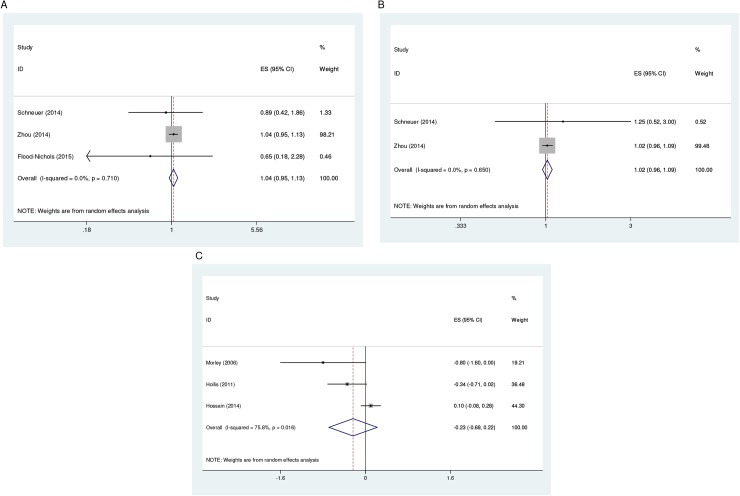
**Forest plot showing the association of vitamin D insufficiency at serum levels of <75 nmol/l with spontaneous abortion (A) and stillbirth (B), and gestational length (C) at low levels.** Abbreviations: ES, Effect Size; CI, Confidence Interval.

Of the four observational studies [25, 26, 31, 33] that investigated spontaneous abortion, three [26, 31, 33] provided estimates for the meta-analysis with first trimester 25(OH)D levels <75 nmol/l found not to be associated with risk of spontaneous abortion (Summary RR = 1.04, 95% CI: 0.95, 1.13) (Table 2, Fig 3A). No evidence of statistical heterogeneity was observed in the meta-analysis. The remaining observational study [25] reported no statistically significant difference in median baseline 25(OH)D levels among women who had miscarriage and those who did not (54 vs. 62 nmol/l, p = 0.14). An intervention study [21] which conducted an intention-to-treat (ITT) analysis also provided evidence on the relationship. In this study, mean baseline 25(OH)D levels of women who experienced pregnancy loss was noted to be lower than women who delivered a live-birth (50.5 vs. 57.8 nmol/L). However, the mean difference was not statistically significant.

#### Stillbirth

Of the four studies that investigated stillbirth, two provided evidence from observational designs [26, 33] with first or second trimester 25(OH)D levels <75 nmol/l found not to be associated with risk of stillbirth (Summary RR = 1.02, 95% CI: 0.96, 1.09) (Table 2, Fig 3B). No evidence of statistical heterogeneity was observed in the meta-analysis. Of the two intervention studies, Hossain et al. [22] reported vitamin D supplementation to have no effect on risk of stillbirth delivery (p = 0.05), whereas Yu et al. [20] recorded the only stillbirth in their study in the control group. Whereas the evidence from Hossain et al. study [22] derives from a *per* protocol analysis, the evidence provided by Yu et al. [20] derives from an ITT analysis.

#### Apgar score

On the relation of vitamin D nutrition status with Apgar score, two studies [19, 22] provided evidence from intervention designs. Sablok et al. [19] reported a positive correlation (r = 0.325, p < 0.001). Hossain et al. [22] also observed 1- and 5-minute Apgar scores to be significantly higher among babies delivered to mothers receiving vitamin D supplementation compared to babies of control mothers (p = 0.03 and p = 0.05, respectively). Both study findings derives from a *per* protocol analysis. Of the three studies that provided evidence from observational designs, Fernandez-Alonso et al. [27] found mothers with first trimester 25(OH)D levels <75 nmol/l to have 40% increased odds of delivering babies with 1-minute Apgar score below 7. Moller et al. [25] and Zhou et al. [26], in contrast, found vitamin D levels not to predict Apgar score. None of the studies reviewed conducted sensitivity analysis that restricted the analysis to term births. With Apgar score positively related to gestational age at delivery, this occurrence raises validity concerns about the findings of the studies reviewed. It was impossible to conduct a meta-analysis owing to the varying measures of association reported by the studies reviewed.

#### Gestational length

Three studies [20–22] provided evidence from intervention designs on the relation of vitamin D nutrition status with gestational length. All the three studies reported no statistically significant difference in mean gestational length between control and intervention groups (p > 0.05, p = 0.17 and p = 0.29, respectively). The findings of two studies [20, 21] derives from an ITT analysis. Two studies [25, 28] provided evidence from observational designs on the relationship with both studies reporting circulating 25(OH)D levels not to be a predictor of gestational length. Two of the intervention studies [21, 22] and one observational study [28] provided estimates for the meta-analysis. No association was found between low third trimester (28–32 weeks) 25(OH)D levels (<28 nmol/l) and short gestational length (Summary ES = -0.24 weeks, 95% CI: -0.69, 0.22) in the meta-analysis (Table 2, Fig 3C). Substantial evidence of statistical heterogeneity was observed in the analysis (*I*^*2*^ = 75.8%). When the analysis was restricted to the intervention studies, again, no association was found (Summary ES = -0.09, 95% CI: -0.524, 0.339) with substantial evidence of statistical heterogeneity observed between the studies (*I*^*2*^ = 77.8%).

### Sources of statistical heterogeneity between included studies

Results of the subgroup analysis are presented in Table 3. For PTB measured at <35–37 weeks and with the application of 25(OH)D cut-off of <75 nmol/l, the summary RR for studies conducted in USA was higher. Moderate evidence of heterogeneity was observed between these studies compared to studies conducted in other locations. The opposite was observed for PTB (<35–37 weeks, <50 nmol/l) and spontaneous abortion.

**Table 3:** Summary relative risk (RR) for the relation of vitamin D insufficiency with pregnancy and birth outcomes stratified according to the study characteristics.

		Random-effects model		Heterogeneity		
Study characteristic	No. of studies	RR	95% CI	Cochran Χ^2^	p value	*I*^*2*^ (%)
**Location**						
PTB (<35–37 weeks, <75 nmol/l)^a^						
USA	4	1.11	0.73, 1.69	5.54	0.137	45.8
Other	3	1.04	1.02, 1.06	0.50	0.778	0.0
PTB (<35–37 weeks, <50 nmol/l)^b^						
USA	1	0.80	0.38, 1.69			
Other	2	0.94	0.68, 1.30	0.13	0.713	0.0
Spontaneous abortion (<75 nmol/l)^a^						
USA	1	0.65	0.18, 2.28			
Other	2	1.04	0.95, 1.13	0.17	0.684	0.0
**Vitamin D assay method**						
PTB (<35–37 weeks, <75 nmol/l)^a^						
LC-MS	3	1.12	0.69, 1.80	5.01	0.082	60.1
CLIA	3	1.04	1.02, 1.06	0.50	0.778	0.0
ELISA	1	0.78	0.17, 3.55			
sPTB (<35–37 weeks, <75 nmol/l)^a^						
LC-MS	3	1.11	0.70, 1.75	4.40	0.111	54.6
ELISA	1	0.78	0.17, 3.55			
PTB (<35–37 weeks, <50 nmol/l)^b^						
LC-MS	2	0.78	0.40, 1.54	0.02	0.882	0.0
HPLC	1	0.95	0.68, 1.33			
Spontaneous abortion (<75 nmol/l)^a^						
CLIA	2	1.04	0.95, 1.13	0.17	0.684	0.0
ELISA	1	0.65	0.18, 2.28			
**Timing of vitamin D assessment**						
PTB (<35–37 weeks, <75 nmol/l)^a^						
First trimester	4	1.10	0.87, 1.39	0.64	0.886	0.0
Second trimester	3	1.15	0.84, 1.57	12.91	0.002	84.5
sPTB (<35–37 weeks, <75 nmol/l)^a^						
First trimester	2	0.84	0.42, 1.70	0.01	0.911	0.0
Second trimester	2	1.17	0.65, 2.12	3.22	0.073	69.0
Spontaneous abortion (<75 nmol/l)^a^						
First trimester	2	0.82	0.43, 1.56	0.18	0.675	0.0
Second trimester	1	1.04	0.95, 1.13			
**Method for assessing gestational age reported**						
PTB (<35–37 weeks, <75 nmol/l)^a^						
Yes	4	1.11	0.73, 1.69	5.54	0.137	45.8
No	3	1.04	1.02, 1.06	0.50	0.778	0.0
PTB (<35–37 weeks, <50 nmol/l)^a^						
Yes	2	0.94	0.68, 1.30	0.13	0.713	0.0
No	1	0.80	0.38, 1.69			
Spontaneous abortion (<75 nmol/l)^a^						
Yes	1	0.65	0.18, 2.28			
No	2	1.04	0.95, 1.13	0.17	0.684	0.0
**Study quality**						
PTB (<35–37 weeks, <75 nmol/l)^a^						
Very high	4	1.18	0.90, 1.55	6.29	0.098	52.3
High	2	1.04	1.02, 1.06	0.14	0.711	0.0
Satisfactory	1	1.12	0.53, 2.37			
sPTB (<35–37 weeks, <75 nmol/l)^a^						
Very high	3	1.11	0.70, 1.75	4.40	0.111	54.6
High	1	0.78	0.17, 3.55			
PTB (<35–37 weeks, <50 nmol/l)^a^						
Very high	2	0.92	0.68, 1.25	0.17	0.681	0.0
Satisfactory	1	0.70	0.14, 3.4			
Spontaneous abortion (<75 nmol/l)^a^						
Very high	1	0.89	0.42, 1.86			
High	2	1.04	0.95, 1.13	0.52	0.469	0.0

Abbreviations: CI, confidence interval; RR, summary relative risk; PTB, preterm birth; sPTB, spontaneous preterm birth; HPLC, High-performance liquid chromatography; CLIA, Chemiluminescence Immunoassay; LC-MS, Liquid chromatography-tandem mass spectrometry; ELISA, Enzyme-linked immunosorbent assay

^a^25(OH)D levels ≥75 nmol/l served as reference.

^b^25(OH)D levels ≥50 nmol/l served as reference.

The summary RR for all PTB outcomes with the exception of PTB (<35–37 weeks, <50 nmol/l), was elevated between studies using LC-MS to assess vitamin status. For spontaneous abortion, the summary RR for the two studies that assayed vitamin D using CLIA method was higher than the estimate of the only study that used ELISA. For PTB (<35–37 weeks, <75 nmol/l) and sPTB (<35–37 weeks, <75 nmol/l), substantial evidence of heterogeneity was observed between the studies using LC-MS assay.

The summary RR for all the outcomes was higher between studies assessing vitamin D status during the second trimester compared to the first trimester. For PTB (<35–37 weeks, <75 nmol/l) and sPTB (<35–37 weeks, <75 nmol/l), again, substantial evidence of heterogeneity between the studies that assessed vitamin D status during the second trimester was noted.

On documentation of the method used for assessing gestational age in the study report, the summary RR for all the outcomes with the exception of spontaneous abortion was elevated between studies that documented this in their report.

In the meta-regression models, vitamin D assay method (β = -0.276, p = 0.049) was the only covariate associated with the heterogeneity observed in the PTB (<35–37 weeks, <75 nmol/l) analysis. For the remaining outcomes, none of the covariates was statistically associated with the observed heterogeneity in the analysis.

### Evidence of publication bias

**Fig 4:**
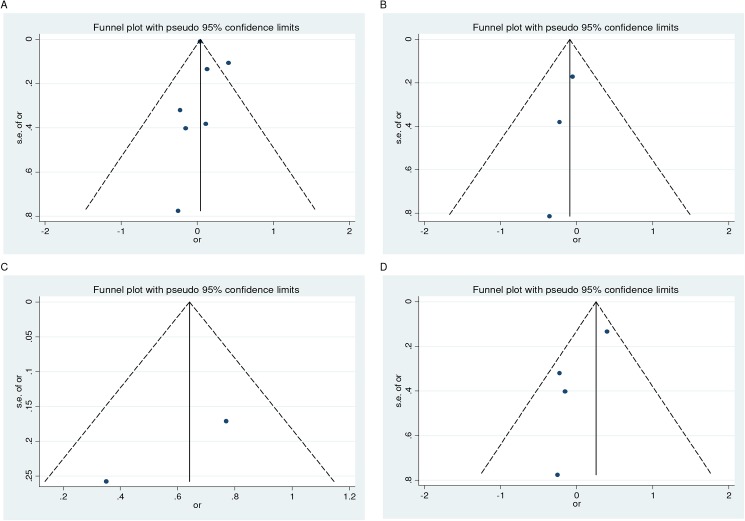
**Funnel plot for the association of PTB (<35–37 weeks) with vitamin D insufficiency at serum levels of <75 nmol/l (A) and <50 nmol/l (B), and PTB (<32–34 weeks) and spontaneous PTB (<35–37 weeks) at serum levels of <75 nmol/l (C and D, respectively)**.

**Fig 5:**
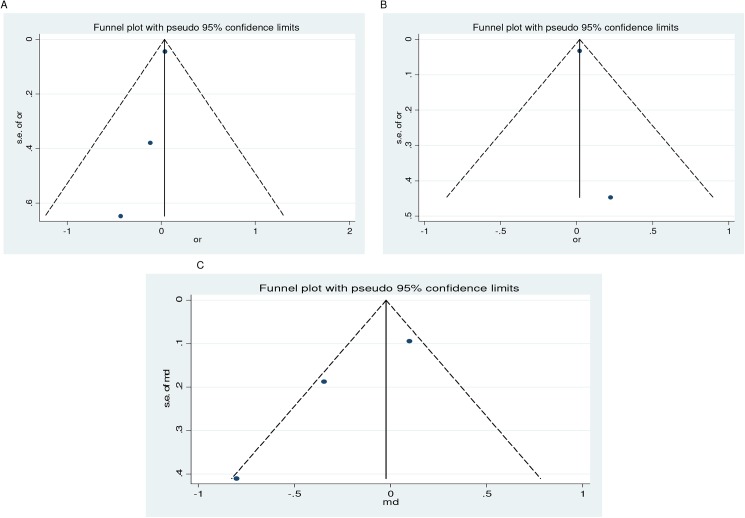
**Funnel plot for the association of vitamin D insufficiency at serum levels of <75 nmol/l with spontaneous abortion (A) and stillbirth (B), and gestational length (C) at low levels**.

**Fig 6:**
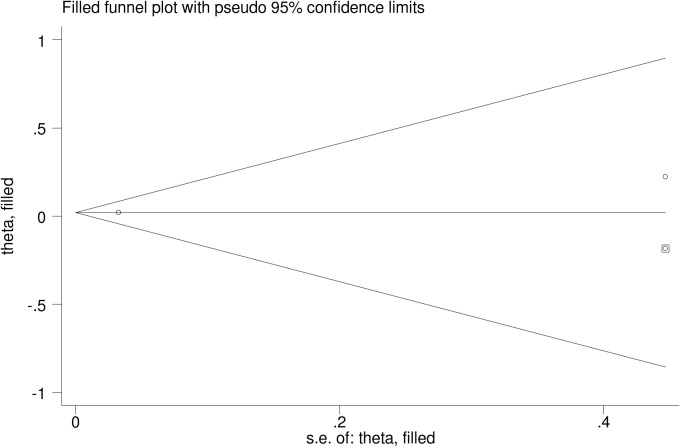
Filled funnel plot for the association between vitamin D insufficiency at serum levels of <75 nmol/l and stillbirth.

The asymmetry observed in the funnel plots suggested evidence of publication bias for the outcomes investigated (Figs 4 and 5). However, with the exception of stillbirth, the Begg's and Egger's test failed to confirm the funnel plot asymmetry observed (Table 4). However, the adjusted estimates for stillbirth did remain unchanged (Table 4). Fig 6 depicts the filled funnel plot for the stillbirth outcome.

**Table 4:** Test for publication bias and adjusted summary relative risk.

	Begg's test		Egger's test			Adjusted summary relative risk^1^		
Outcome	z	p value	Bias coefficient	95% CI	p value	No. of studies	RR	95% CI
Gestational length	-1.57	0.117	-3.303	-15.001, 8.396	0.173			
PTB (<35–37 weeks, <75 nmol/l)	-0.15	0.881	0.424	-1.338, 2.185	0.563			
PTB (<32–34 weeks, <75 nmol/l)	-1.00	0.317	-4.846	-	-			
sPTB (<35–37 weeks, <75 nmol/l)	0.00	1.00	-1.752	-4.783, 1.279	0.131			
PTB (<35–37 weeks, <50 nmol/l)	-0.52	0.602	-0.572	-2.733, 1.589	0.184			
Spontaneous abortion (<75 nmol/l)	-1.57	0.117	-0.631	-2.641, 1.379	0.156			
Stillbirth (<75 nmol/l)	1.00	0.317	0.490	-	-	3	1.02	0.96, 1.09

Abbreviations: CI, confidence interval; RR, summary relative risk; PTB, preterm birth; sPTB, spontaneous preterm birth.

^1^Estimated from random-effects model.

## Discussion

### Summary of findings

We systematically reviewed 18 studies that investigated the association of vitamin D nutritional status with pregnancy and birth outcomes. For spontaneous abortion and stillbirth, from the limited evidence uncovered, vitamin D insufficiency was not associated with risk of these outcomes. However, we found sufficient evidence from observational studies linking vitamin D insufficiency with PTB. Serum 25(OH)D levels <75 nmol/l was associated with 13% and 83% increased risk of PTB measured at <35–37 weeks and <32–34 weeks, respectively. An inverse dose-response relation was noted for both PTB outcome. Serum 25(OH)D levels <75 nmol/l was also associated with 11% increased risk of spontaneous PTB measured at <35–37 weeks, with a dose-response relation also noted. Decreased vitamin D levels during pregnancy was not associated with short gestational length (ES = -0.24, 95% CI: -0.69, 0.22). The limited evidence available on vitamin D nutritional status and Apgar score was conflicting and controversial.

### Validity issues

We performed a comprehensive search of PubMed and Scopus databases, which indexes majority of scientific journals. We searched these databases using a well-defined search strategy that involved the use of both controlled vocabulary and text words, and with no language restrictions applied. We also screened the reference list of all included studies and previous related reviews of the topic.

We included only longitudinal studies which enables an objective assessment of temporality, thereby eliminating doubts about the observed associations. The inclusion of only studies that applied direct methods in assessing vitamin D nutritional status also minimizes misclassification bias and helps strengthen our findings. We have also detailed the methodological limitations of the included studies, pointing out the implications that these limitations have for the quality of the evidence reported by the studies. For the meta-analysis, we conducted a sensitivity analysis by restricting the analysis to very high quality studies to assess the robustness of our results. For PTB measured at <35–37 weeks, when a serum 25(OH)D cut-off level of 75 nmol/l was applied, the estimates from the sensitivity analyses was attenuated.

We investigated publication bias to account for unpublished studies. However, the results from the Begg's and Egger's test failed to confirm the funnel plots asymmetry observed in almost all of the analyses. This inconsistency could be attributed to numerical instability owing to the inability to include in the meta-analysis, studies that provided no estimates for the meta-analysis and were subsequently reviewed qualitatively. We conducted dose response meta-analyses to provide insights into causality. We also conducted sub-group analyses and meta-regression to elaborate the observed heterogeneity in the analysis. However, it must be emphasized that, the Cochran X^2^ test performed has low statistical power to detect heterogeneity if the meta-analyses include few studies. As a result, we complemented the Cochran X^2^ test by computing the I^2^ statistic, which quantifies the impact of heterogeneity and assess inconsistency, and does not depend on the number of studies. That notwithstanding, results of the statistical heterogeneity reported for the dose response and sub-group analyses should be interpreted with caution owing to the small number of studies.

### Synthesis of findings with previous knowledge

Our review found vitamin D insufficiency to be associated with both early PTB (<32–34 week) and late PTB (<35–37 weeks). Two previous reviews [8,11] found no association of vitamin D insufficiency with PTB. Harvey et al. [11] indicated that the evidence evaluated does not support the use of maternal supplementation to prevent PTB. The consistency of the results of the studies we evaluated, the precision of the effect estimates, the significant dose-response relation noted in the analysis, and the known biologic plausibility (which derives from vitamin D's immunomodulatory roles and prevention of intrauterine infection), do possibly implicate vitamin D insufficiency in PTB occurrence.

Vitamin D has immunomodulatory roles during pregnancy that enable successful implantation by attenuating decidual T-cell function [37,38]. The immunomodulatory effects of vitamin D have led to speculation that vitamin D could act as an immune regulator during implantation and hence may play an important role in reproductive capacity [39]. Vitamin D also regulates key target genes associated with proper implantation of the placenta [40] and is also important for pregnancy maintenance through its relation to calcium metabolism in the myometrium [41–43]. Vitamin D is documented to have a direct role in the production of antimicrobial peptides such as cathelicidin through activation of up-regulated vitamin D receptors in the presence of 25(OH)D as substrate and may play an important role in preventing infection during pregnancy or early childhood [44–47]. According to Romero et al. [48], the biological mechanisms through which intrauterine infections lead to preterm labour are related to activation of the innate immune system. Thorne-Lyman and Fawzi [8], stated that the influence of vitamin D status on bacterial infections during pregnancy is plausible and is supported by the consistency of the results of studies associating vitamin D insufficiency with bacterial vaginosis during pregnancy.

## Conclusions

In conclusion, our systematic review and meta-analysis found vitamin D insufficiency to be associated with PTB, but not with spontaneous abortion and stillbirth. However, caution is required in inferring causality owing to the limited and weak experimental evidence available. The evidence on vitamin D nutrition and low Apgar score was conflicting and controversial.

Clinical decision making is largely based on experimental evidence and as a result we recommend the conduct of more intervention studies in diverse geographical settings to further evaluate the benefits of vitamin D supplementation during pregnancy on fetal health and the prevention of adverse pregnancy and birth outcomes. Future studies should also clarify the exact 25(OH)D level that can be deemed sufficient for improved maternal and perinatal health owing to the lack of consensus in the literature.

## Supporting information

S1 Appendix PRISMA checklist. (DOC)
